# Validating a targeted next-generation sequencing assay and profiling somatic variants in Chinese non-small cell lung cancer patients

**DOI:** 10.1038/s41598-020-58819-5

**Published:** 2020-02-07

**Authors:** Ruirui Jiang, Bo Zhang, Xiaodong Teng, Peizhen Hu, Sanpeng Xu, Zuyu Zheng, Rui Liu, Tingdong Tang, Feng Ye

**Affiliations:** 1 Laboratory of Pathology, West China Hospital, Sichuan University, Chengdu, 610041 China; 2 Department of Pathology, West China Hospital, Sichuan University, Chengdu, 610041 China; 3 Department of Pathology, The First Hospital of Lanzhou University, Lanzhou, 730000 China; 4 Department of Pathology, Peking University Third Hospital, Beijing, 100191 China; 5 Department of Pathology, The First Affiliated Hospital, Zhejiang University, Hangzhou, 310003 China; 6 Department of Pathology, Xijing Hospital, Air Force Medical University, Xian, 710032 China; 7 Institute of Pathology, Tongji Hospital, Tongji Medical College, Huazhong University of Science & Technology, Wuhan, 430030 China; 8 Singlera Genomics Inc., Shanghai, 201318 China

**Keywords:** Non-small-cell lung cancer, Molecular medicine

## Abstract

Non-small cell lung cancer (NSCLC) is featured with complex genomic alterations. Molecular profiling of large cohort of NSCLC patients is thus a prerequisite for precision medicine. We first validated the detection performance of a next-generation sequencing (NGS) cancer hotspot panel, OncoAim, on formalin-fixed paraffin-embedded (FFPE) samples. We then utilized OncoAim to delineate the genomic aberrations in Chinese NSCLC patients. Overall detection performance was powerful for mutations with allele frequency (MAF) ≥ 5% at >500 × coverage depth, with >99% sensitivity, high specificity (positive predictive value > 99%), 94% accuracy and 96% repeatability. Profiling 422 NSCLC FFPE samples revealed that patient characteristics, including gender, age, lymphatic spread, histologic grade and histologic subtype were significantly associated with the mutation incidence of *EGFR* and *TP53*. Moreover, RTK signaling pathway activation was enriched in adenocarcinoma, while PI(3)K pathway activation, oxidative stress pathway activation, and TP53 pathway inhibition were more prevalent in squamous cell carcinoma. Additionally, novel co-existence (e.g., variants in *BRAF* and *PTEN*) and mutual-exclusiveness (e.g., alterations in *EGFR* and *NFE2L2*) were found. Finally, we revealed distinct mutation spectrum in *TP53*, as well as a previously undervalued *PTEN* aberration. Our findings could aid in improving diagnosis, prognosis and personalized therapeutic decisions of Chinese NSCLC patients.

## Introduction

Lung cancer is the leading cause of cancer-associated mortality worldwide. Up to 90% lung cancer is non-small cell lung cancer (NSCLC)^1^. NSCLC is usually diagnosed at the advanced stage with limited therapeutic options (e.g., surgery, radiotherapy and chemotherapy)^1^.

Conventional molecular detection techniques, such as fluorescence *in situ* hybridization and Sanger sequencing, only detect a limited number of biomarkers^2–4^. The advent of next-generation sequencing (NGS) has broadened the landscape of genetic aberrations^5–7^, making it possible to implement targeted treatment tailored for specific mutations in individual patients^1,8,9^.

Lung cancer is characterized with complex genomic aberrations^10^. The most frequently mutated genes in NSCLC include *EGFR* (Epidermal growth factor receptor), *TP53* (Tumor Protein p53), *KRAS* (Kirsten rat sarcoma viral oncogene) and *PIK3CA* (Phosphatidylinositol 3-kinase). In addition, Genetic mutations detected in NSCLC are complicated by patient demographic, racial, clinical and pathological characteristics^1,8,9,11^. For instance, the mutational frequency of *EGFR* can vary from 10% in Western populations to 30% in Asians, and *KRAS* mutation incidence in Western populations is approximately 18-26% versus 3.8-8% in Asians^12^, and within Asian populations, the distinct ethnicities bear *EGFR* aberrations at a rate ranging from 19.6% to 40.1%^11^. A comprehensive database of genetic aberrations in large cohorts of NSCLC patients is thus required for clinical interpretation of variants^1,10^. Subtyping of lung cancer based on specific molecular markers and understanding the potential association of patient genetic alterations with clinicopathological characteristics may help improve early NSCLC diagnosis, prevent NSCLC incidence, and guide more beneficial treatments^6,13^. For example, NSCLC patients with *EGFR* mutations and *ALK* (Anaplastic lymphoma kinase) gene rearrangement will benefit from targeted therapy of EGFR-TKIs (Tyrosine kinase inhibitors) (such as erlotinib and afatinib)^14^ and ALK inhibitors (crizotinib, ceratinib, etc.)^15^, respectively.

The tumor samples are typically preserved as formalin-fixed paraffin-embedded (FFPE), which causes DNA fragmentation and crosslinking. The reduced DNA quality in FFPE samples, along with normally low mutation frequency and tumor heterogeneity, necessitates higher sequencing coverage for reliable mutation calling^16–18^. Thus, targeted sequencing–enriching the regions of interest prior to sequencing–is more reliable and cost-effective compared with genome-wide sequencing^7,19^. However, stringent validation of NGS-based cancer detection in FFPE specimens for clinical testing is still very rare.

Here we developed and validated an amplification-based cancer hotspot panel, OncoAim (Singlera Genomics, Shanghai, China). The panel covers the mutational hotspots of 59 genes implicated in common cancers including lung cancer. We evaluated the detection performance of OncoAim on single nucleotide variants (SNVs) and short insertions and deletions (INDELs) in FFPE specimens using Ion Torrent PGM (Personal Genome Machine) platform. Our results established that OncoAim was suitable for clinical application in FFPE samples. This assay, along with our findings in Chinese NSCLC patients could aid in improving the diagnosis, prognosis and personalized therapeutic decisions of Chinese NSCLC patients.

## Results

### Performance validation of OncoAim on SNVs

For SNVs detection performance (sensitivity and specificity), we used 4 individual reference standards (i.e., HD200, HD300, HD301, and HD802) and 4 mixed reference standards (i.e., HD300-HD706, HD802-HD260, HD301-HD706, and HD200-HD706). These references together possessed 54 known SNVs with MAF ranging from 1% to 70.0% (Table S3a). The median sequencing coverage depth of these 8 libraries were between 1025× and 1469× (Table S3b). The overall SNVs detection performance was high: >99% (40/40) of expected SNVs with MAF ≥ 5% were successfully detected, and 79% (11/14) of alterations at MAF < 5% were also identified (Table S3c). In addition, high specificity was maintained with a PPV > 99% (51/51).

**Figure 1:**
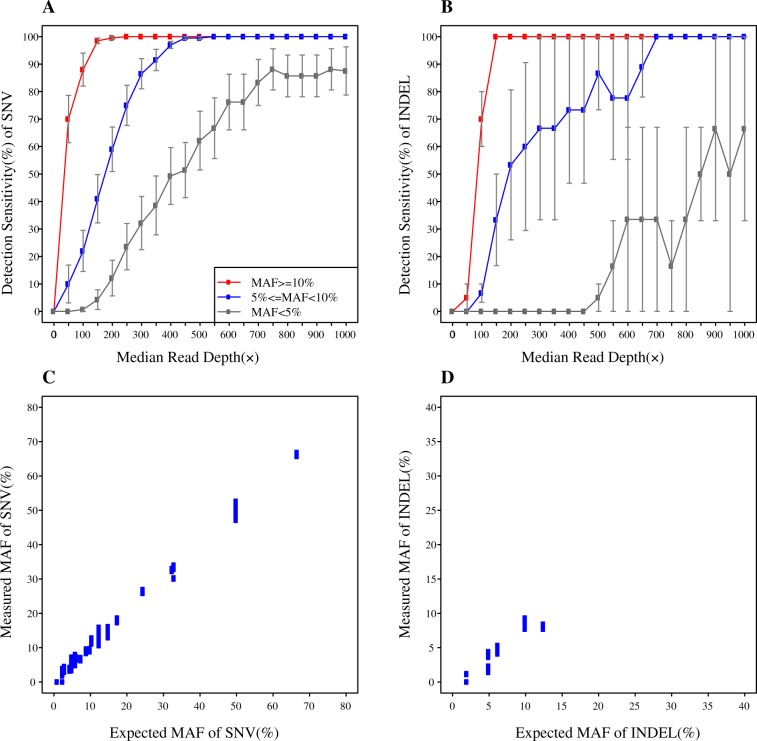
SNV and INDEL detection performance of OncoAim (panels A,C for SNV; panels B,D for INDEL). (**A**,**B**) Detection sensitivity was plotted against the median sequencing coverage depth. Error bars, standard error of the mean (SEM). (**C**,**D**) Detected allele frequencies were in concordance with those expected in reference standards.

We then evaluated the impact of sequencing coverage depth (50-1000×) and MAF on SNV detection by randomly subsampling data (Tables S3a,c). Detection sensitivity steadily increased as coverage depth increased in all MAF sections (<5%, 5-10%, and ≥10%) (Fig. 1A), and high sensitivity was obtained up to 500× median coverage depth (Fig. 1A). At 500×, >99% (400/400) of SNVs with expected MAF ≥ 5% were successfully detected. Furthermore, PPV remained high (>99%) across the full coverage depth range (Table S4). We observed a high correlation between expected and observed MAFs (Fig. 1C), highlighting the robust quantitative characteristics of OncoAim test.

### Performance validation of OncoAim on INDELs

For INDELs detection validation, we used 3 individual reference standards (i.e., HD200, HD300, and HD802) and 3 mixed reference standards (i.e., HD200-HD706, HD300-HD706, and HD802-HD260). These references contain 7 known INDELs with a wide range of MAF and INDELs length (3-15 bp) (Table S5a). The average sequencing coverage depth across six samples was 1232× (Table S5b). We observed high overall detection performance: >99% (5/5) of expected INDELs with MAF ≥ 5% were successfully detected (Table S5c). The PPV was >99% (5/5).

The effects of sequencing coverage depth and MAF on INDELs detection performance were assessed by subsampling data (Table S5a,c). The INDELs detection sensitivity increased with increasing coverage depth at MAF ≥ 5%, and high sensitivity was observed up to 700× median coverage depth. At 700×, >99% of INDELs at MAF ≥ 5% (15/15) were successfully detected. We observed a fluctuating trend of detection sensitivity with varying coverage depth at low MAF (<5%) (Fig. 1B). Moreover, PPV achieved was 85–100% across the full coverage depth (Table S6). The correlation between measured and expected MAF for INDELs was high (Fig. 1D).

### Concordance between OncoAim NGS test and orthogonal ARMS-PCR test

**Figure 2:**
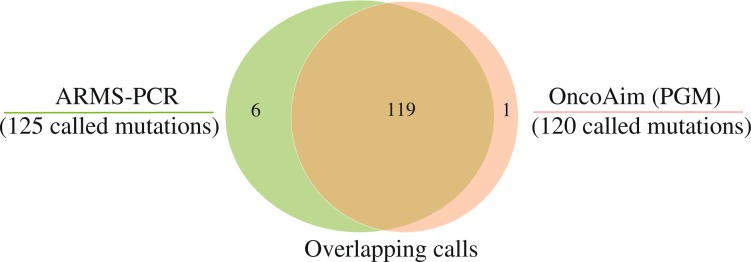
Concordance between OncoAim and ARMS-PCR approaches on FFPE specimens. Overlap of positive variant calls by NGS (OncoAim) and ARMS-PCR methods at tested sites in 253 FFPE clinical cancer specimens. Note: among the 119 common variants, 106 variants with MAF ≥ 5% was called by OncoAim when the bioinformatics analysis pipeline used 5% as cut-off for variant calling. The other 13 variants missed by OncoAim analysis pipeline (5% cut-off) had MAF < 5%, which were detected but filtered out. These 13 variants were also counted as detected by OncoAim here for better comparison of two methods.

We tested the *EGFR* mutation status in 253 FFPE NSCLC specimens (a subset of 452 total cases) using both OncoAim and ARMS-PCR (Amplification refractory mutation system-Polymerase Chain Reaction) technique. In total, two methods detected 126 *EGFR* mutations with 94% concordance (119 aberrations were detected by both platforms) (Fig. 2). Six variants were detected by ARMS-PCR but not by NGS (Table S7), likely due to the low MAF of these variants and/or tumor heterogeneity. NGS, but not ARMS-PCR, detected one mutation that was confirmed as TP by Sanger sequencing (Table S7).

### Reproducibility of NGS test on FFPE samples

We assessed the precision (reproducibility and repeatability) of OncoAim test with 3 FFPE tumor samples that possess 5 known alterations (SNVs and INDELs including KRAS p.G12V, KRAS p.G12D, TP53 p.R248W, TP53 p.R333fs*12, EGFR p.L858R) in total. Among these 5 known alterations, mutations of KRAS and EGFR were detected previously by ARMS-PCR method, and TP53 mutations were detected previously by NGS (Illumina Miseq platform). The samples were analyzed 5 times in 3 different experiments to evaluate the inter-run and intra-run reproducibility. Concordance between replicates was 96% (Table S8), with no significant differences between inter- and intra-run replicates, demonstrating the robustness of the NGS test.

### Mutational profiling of Chinese NSCLC patients

A total of 422 cases of the 452 FFPE samples was successfully sequenced for subsequent variation analysis (Fig. S1), and 30 cases were unsuccessful for several reasons, such as poor DNA quality, low library concentration, and low median read coverage and uniformity. We then utilized OncoAim to profile the mutational landscape of 422 Chinese NSCLC patients. The clinicopathological characteristics of patients were summarized in Table 1.

**Table 1:** Clinicopathological characteristics of NSCLC samples analyzed in this study.

Characteristics	Number of patients
Total cases	422
Gender	
Female	198
Male	191
NA	33
Age	
(17, 45)	116
(45, 65)	190
(65, 86)	83
NA	33
Smoking	
No	82
Yes	13
NA	327
Primary vs. Metastatic tumor	
Primary	190
Metastatic	22
NA	210
Lymphatic spread	
No	158
Yes	73
NA	191
Tumor site	
Left_lung	26
Right_lung	19
NA	377
Histologic grade	
Badly differentiated	51
Moderately differentiated	136
Well differentiated	83
NA	152
Histologic subtype	
Adenocarcinoma	217
Adenosquamous carcinoma	4
Large cell carcinoma	1
Squamous cell carcinoma	56
NA	144

NA, not available.

**Figure 3:**
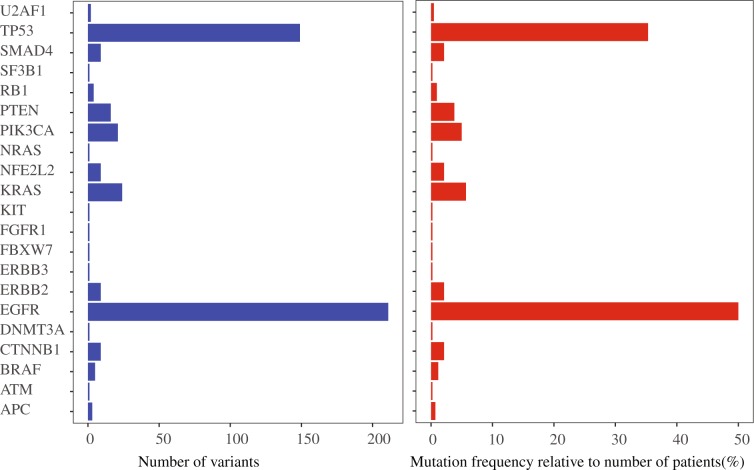
Mutated genes, the number of aberrations and the mutation frequency relative to the number of patients recruited in this study.

**Figure 4:**
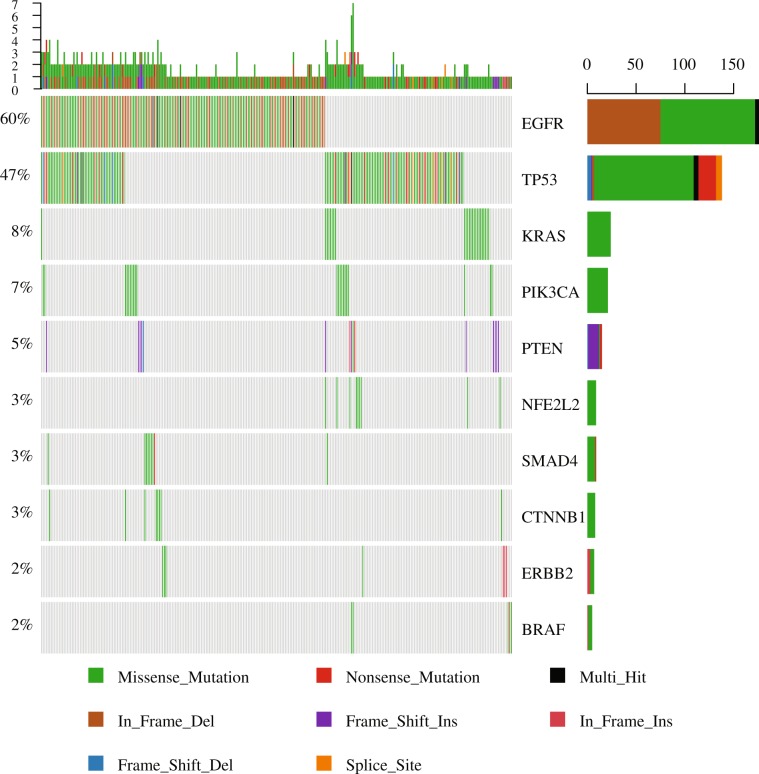
Oncoplot depicting the top 10 most frequently mutated genes. The frequency is calculated by dividing the number of variants in the specific gene by the number of patients with at least one mutation identified. Top panel shows the number of aberrations detected in each sample. The right panel displays number of variants in each gene.

Approximately 70% (295) of 422 FFPE samples possessed at least 1 mutation. In total, 479 mutations in 21 genes were identified (Fig. 3). The gene mutation frequency, calculated by dividing the number of mutations in individual gene by the number of patients, ranged from 0.24% to 50%. The top 5 most frequently mutated genes were *EGFR* (211, 50%), Tumor protein p53 (*TP53*) (149, 35%), *KRAS* (24, 5%), phosphatidylinositol-4,5-bisphosphat3-kinase, catalytic subunit alpha (*PIK3CA*) (21, 5%), and phosphatase and tensin homolog (*PTEN*) (16, 4%) (Fig. 3). Some patients had more than one *EGFR* mutation, as 211 *EGFR* alterations were detected from 176 samples (Fig. 4). In addition, one patient acquired two *PTEN* mutations.

Missense mutation was the most prevalent form in almost all the top 10 mutated genes, with *PTEN* as the exception in which in-frame-insertion was the major type (Fig. 4). In-frame-deletion and nonsense mutation ranked in 2^nd^ place in *EGFR* and *TP53* mutations, respectively. All mutations in *KRAS*, *PIK3CA*, nuclear factor erythroid-derived 2-like 2 (*NFE2L2*) and Catenin, beta-1 (*CTNNB1*) were missense type (Fig. 4).

### Mutational hotspots in the top 5 most frequently mutated genes

**Figure 5:**
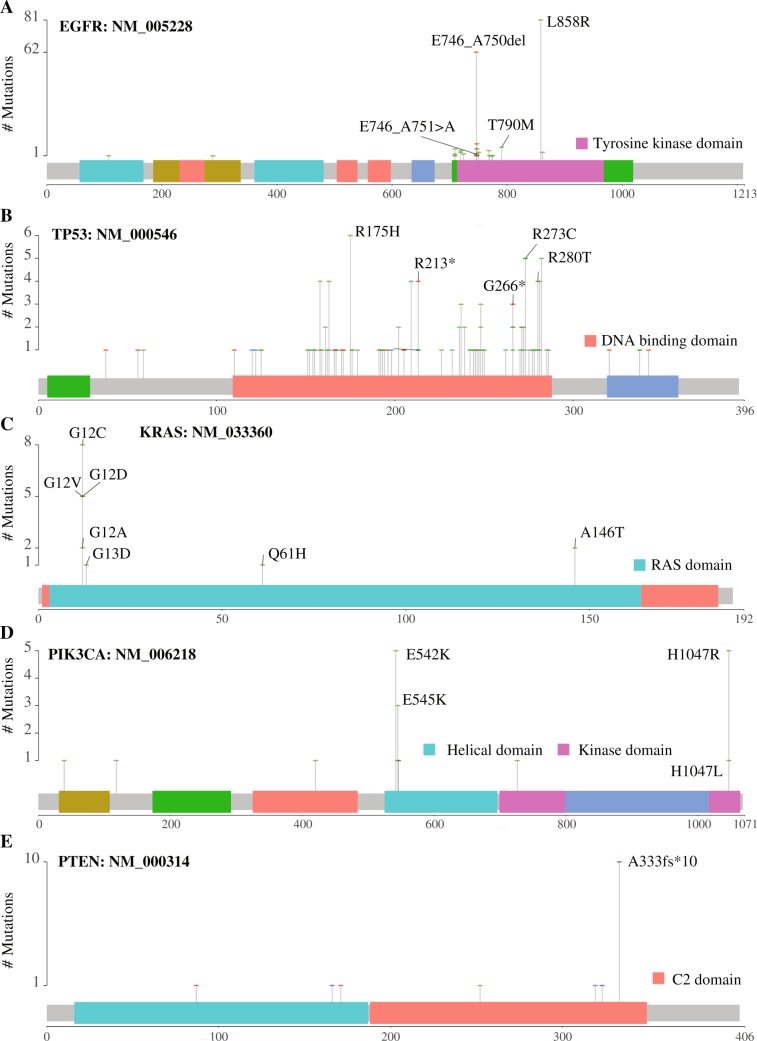
Mutational hotspots of the top 5 most frequently mutated genes including *EGFR* (**A**), *TP53* (**B**), *KRAS* (**C**), *PIK3CA* (**D**), and *PTEN* (**E**). All the variants on *KRAS* were labelled. For other genes, only the most prevalent and critical variants were marked. The protein domains harboring these mutational hotspots were noted.

The mutational hotspots of the 5 most frequently mutated genes are summarized in Fig. 5.

*EGFR* is a transmembrane tyrosine kinase belonging to HER/erbB protein family. Its activation can activate downstream PI3K-AKT-mTOR and RAS-RAF-MEK-ERK signaling pathways^20^. The most observed mutations in *EGFR* were L858R missense mutation (exon 21) and an in-frame deletion from E746 to A751 (exon 19) (Fig. 5A), consistent with previous reports^21^. These mutations confer increased sensitivity to both first-generation and second-generation EGFR Tyrosine kinase inhibitors (TKIs)^21–23^. Notably, the T790M variant, which is often induced by TKIs treatment and thus a resistance marker to first-generation TKIs (erlotinib and gefitinib), was detected 8 times (approximately 4% of the 211 *EGFR* mutations) (Fig. 5A). T790M mutation has been suggested as the primary mutation in some NSCLC patients^24^.

*TP53* is one of the best-known tumor suppressor genes. Since *TP53* protein forms a tetramer for binding to cis-elements, some of its missense mutants may have dominant-negative effects on wild-type *TP53* via oligomerization^25,26^. We noticed 73.83% of *TP53* aberrations were the missense type. *TP53* mutations were clustered in the DNA binding domain (amino acid 101-300), with R273 (7.09%), G266 (5.67%), R213 (4.96%), R175 (4.26%), and R280 (4.26%) as the top 5 most frequently mutated sites (Fig. 5B). Two of these 5 residues, R273 and R175, belong to the previously reported 6 mutational hotspots of *TP53* (i.e., R175, G245, R248, R249, R273 and R282)^26^. Among these 6 reported residues, R248 and R282 also had high frequency in the present study, both at 3.55%. However, G245 and R249 showed up at low frequency, 1.42% and 0.71%, respectively, indicating they were not prevalent in Chinese NSCLC patients. R213* nonsense mutation was the most common nonsense mutant (Fig. 5B), consistent with a previous study^26^. However, the high mutation frequency of R213* in our study has not previously been reported. This result, together with the observed high mutation frequency of G266 and R280, might represent the unique features of *TP53* mutations in Chinese NSCLC patients.

*KRAS* is a member of the small GTPase superfamily whose activating mutations can constitutively activate downstream RAF-mediated signaling pathway, leading to uncontrolled cell proliferation^27^. NSCLC patients carrying KRAS mutations develop primary resistance to EGFR-targeted drugs such as cetuximab and gefitinib^28^. G12 was the most dominant mutated residue in our study (Fig. 5C), consistent with other reports^1^. Lung cancer patients with KRAS G12C and G12V mutations have shorter PFS (progression-free survival) compared with those patients carrying other KRAS mutations or KRAS wild type^29^.

*PIK3CA* is one of the catalytic subunits of phosphatidyl 3-kinases (PI3K), modulating various cellular processes^30^. We identified E542, E545 (exon 10, the helical domain) and H1047 (exon 21, the kinase domain) as the most frequently mutated sites (Fig. 5D). These *PIK3CA* mutations are regarded as oncogenic variants and can be targeted for drug development^31^.

*PTEN* acts as a tumor suppressor, and its best-known role is to antagonize the PI3K signaling pathway through its lipid phosphatase activity^32^. A333*fs10 (COSM5346961) in the C2 domain was the most frequently observed *PTEN* aberration in our cohort (Fig. 5E). The C2 domain is crucial for anchoring the *PTEN* catalytic domain onto the membrane.

### Association of patient clinicopathological characteristics with mutations

Associations of mutations with the following clinicopathogical characteristics, gender, age, smoking etc were evaluated (Table 2).

**Table 2:** Association Analysis of Clinicopathological Characteristics with Mutations.

Characteristics	Gene	Group	Wild type	Mutant	Mutation frequency, %	P value
Gender	EGFR***	Male	139	59	29.80	1.40E-11
		Female	69	122	63.87	
	TP53***	Male	103	95	47.98	8.90E-08
		Female	149	42	21.99	
	PTEN*	Male	186	12	6.06	3.20E-02
		Female	188	3	1.57	
Age	EGFR**	(17,45)	77	39	33.62	1.40E-03
		(45,65)	86	104	54.74	
		(65,86)	45	38	45.78	
	TP53***	(17,45)	97	19	16.38	1.00E-06
		(45,65)	107	83	43.68	
		(65,86)	48	35	42.17	
Smoking	KRAS*	Yes	11	2	15.38	1.70E-02
		No	82	0	0.00	
	PIK3CA*	Yes	11	2	15.38	1.70E-02
		No	82	0	0.00	
	BRAF	Yes	13	0	0.00	1.00E + 00
		No	79	3	3.66	
Tumor type	EGFR*	Metastatic	9	13	59.09	1.60E-02
		Primary	130	60	31.58	
Lymphatic spread	EGFR**	Yes	22	51	69.86	4.30E-03
		No	80	78	49.37	
	TP53*	Yes	48	25	34.25	1.30E-02
		No	128	30	18.99	
Histologic grade	EGFR***	Poorly	29	22	43.14	4.90E-06
		Moderately	51	85	62.50	
		Well	59	24	28.92	
	TP53***	Poorly	33	18	35.29	4.70E-04
		Moderately	77	59	43.38	
		Well	68	15	18.07	
Histologic variant	EGFR***	ACA	119	98	45.16	9.10E-07
		SCC	50	6	10.71	
	TP53***	ACA	164	53	24.42	1.60E-08
		SCC	19	37	66.07	
	PIK3CA*	ACA	209	8	3.69	1.80E-02
		SCC	49	7	12.50	
	NFE2L2**	ACA	217	0	0.00	1.60E-03
		SCC	52	4	7.14	
Tumor site	/	Left lung	/	/	/	/
		Right lung				

Except for BRAF gene in smoking status, only statistically significant mutated genes were shown. For two nominal variables, two-sided Fisher’s exact test was performed. For analysis involving more than 2 groups (age and histologic grade), following two-sided Fisher’s exact test (P value was provided in the table), pairwise comparison was done with P value adjusted with Bonferonni correction. The adjust P value for statistically significant pair was specified in the main text where appropriate. “/” for tumor site indicates no significantly differentially expressed genes. ACA, Adenocarcinoma; SCC, Squamous cell carcinoma. *P ≤ 0.05; **P ≤ 0.01; ***P ≤ 0.001.

Mutations in lung cancer can be associated with gender^1^. We observed that female patients had significantly higher frequency of *EGFR* mutations (*P* = 1.41E-11, Fisher’s exact test) but a significantly lower mutation frequency of *TP53* (*P* = 8.912E-08) and *PTEN* (*P* = 3.2E-2) (Table 2 and Fig. S2), consistent with previous reports^5,33,34^. Interestingly, *PTEN* mutations were more prevalent in male patients.

Compared with the middle-aged group (45-65 years old), the young-aged group (18-45 years old) had a significantly lower *EGFR* mutation rate (*P* = 1.16E-03, adjusted by Bonferroni’s correction) (Table 2 and Fig. S3), which agrees with other studies^35,36^. Mutations in *TP53* were less frequent in the young-aged group as opposed to both the middle-aged and older group (>65 years), with *P* values of 2.45E-06 and 2.59E-04, respectively (Table 2).

Smoking is a crucial risk factor for lung cancer^1,9^. The increased frequency of *KRAS* mutation in smokers (*P* = 1.7E-02) (Table 2 and Fig. S4) is consistent with other reports^24,27^. We observed a positive correlation of *PIK3CA* aberrations with smoking status (*P* = 1.7E-02) (Table 2), inconsistent with a previous study in Japanese lung cancer patients^37^. This discrepancy might result from ethnic variations.

Lymph node metastasis is a strong independent predictor of poor prognosis^38^. The lung tumors with lymphatic spreading were enriched with mutations in *EGFR* (*P* = 4E-03) and *TP53* (*P* = 1.3E-02) (Table 2 and Fig. S5), indicating that tumors with *EGFR* or *TP53* mutations were more likely to develop metastatic tumors and might have poorer prognosis. These findings were consolidated by a previous report showing that *EGFR* expression level is higher in lung cancer patients with lymph node metastasis^39^.

Lung cancer, at later developing stages, tends to spread to other body parts forming metastatic lung tumors. Compared with primary lung tumors, metastatic lung tumors had higher mutational frequency of *EGFR* (*P* = 1.6E-02, Table 2 and Fig. S6). Cautious screening and monitoring of potential metastasis should be performed for patients with primary lung tumors that harbor *EGFR* driver mutations.

Histologic grade of lung cancer is an independent predictor of patient survival^40^. We observed that the well differentiated tumor has significantly lower mutation frequency of *EGFR* (*P* = 5.25E-06, adjusted by Bonferroni’s correction) and *TP53* (*P* = 3.54E-04, adjusted by Bonferroni’s correction) than moderately differentiated tumors (Table 2 and Fig. S7). The well-differentiated tumors also have lower *EGFR* and *TP53* mutation frequencies compared with poorly differentiated tumors, but these differences did not reach a significant level (*P* = 3.99E-01 and 1.14E-01, adjusted by Bonferroni’s correction, respectively). The low mutation frequency of *EGFR* and *TP53* in well-differentiated tumors might represent a marker for better prognosis.

Adenocarcinoma (ACA) and squamous cell carcinoma (SCC) are the two major histological subtypes of NSCLC, and they are featured with distinct gene expression profiles with different clinical implications^41^. While *EGFR* mutations were more prevalent in ACA (*P* = 9.12E-07), the mutations of *TP53*, *PIK3CA*, and *NFE2L2* were enriched in SCC, with *P* values of 1.56E-08, 1.8E-02, and 2E-03, respectively (Table 2 and Fig. S8). These results indicated that RTK (Receptor Tyrosine Kinase) signaling pathway activation was enriched in ACA, whereas PI(3)K pathway activation, oxidative stress pathway activation, and *TP53* pathway inhibition were more prevalent in SCC. Although such correlations for each gene have been previously reported^42–45^, this study is the first to reveal the histological subtype association of these 4 genes in one lung cancer cohort study. The transcription factor *NFE2L2* plays central roles in modulating the expression of genes involved in antioxidant and stress-response^46^, and its D29Y, D29H and R34Q variants (on exon 2) detected in this study might indicate poor prognosis of lung cancer^42,47^.

We didn’t find differentially mutated genes in different tumor sites (left lung vs. right lung) (Table 2 and Fig. S9).

### Mutually exclusive and co-occurring variants in NSCLC

**Figure 6:**
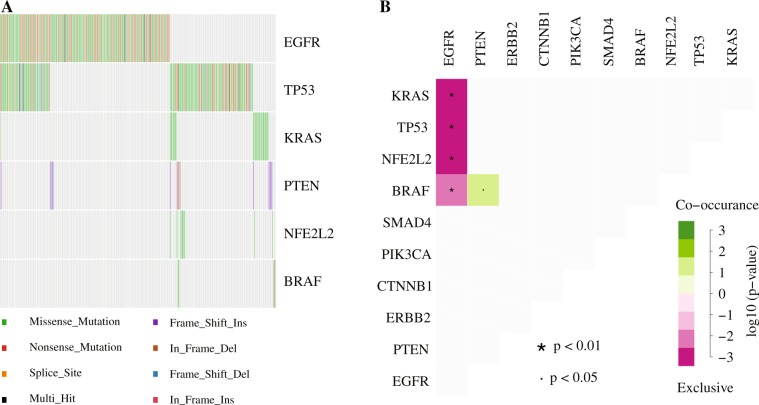
Mutual exclusiveness and co-existance of variants. (**A**) Oncostip plot displayed the distribution of all the mutations across the samples containing at least one aberration in *EGFR*, *TP53*, *KRAS*, *PTEN*, *NFE2L2*, and *BRAF*. (**B**) Mutually exclusive gene pairs or co-occurring gene pairs were depicted.

Our data demonstrated *EGFR* was mutated in a mutually exclusive fashion with *KRAS*, *TP53*, *NFE2L2*, and B-Raf proto-oncogene, serine/threonine kinase (*BRAF*) (Fig. 6). The mutual exclusiveness between *EGFR* and *KRAS* or *BRAF* has been well-known^20,48,49^. This is the first evidence showing *NFE2L2* mutations did not coexist with *EGFR* mutations, consolidating the oncogenic nature of *NFE2L2* mutations^47^. Interestingly, *TP53* mutations were mutually exclusive with *EGFR* mutations to a significant level (Fig. 6). This new observation was, to some extent, supported by the finding that *RAS* and *TP53* show mutual exclusiveness in acute myeloid leukemia^50^, suggesting that inactivating *TP53* alone may be sufficient for lung cancer cells to proliferate and circumvent apoptosis.

We observed that *PTEN* mutations tended to coincide with *BRAF* mutations (Fig. 6). The *PTEN* mutations result in activation of the PI(3)K pathway. Studies in lung cancer have not been reported on the co-occurrence of mutations in *PTEN* and *BRAF*, but the *PIK3CA* mutations have been associated with *BRAF* mutations^51^. In melanoma, however, loss-of-function *PTEN* mutations and *BRAF* activation mutations coexisted^52^. These findings indicate that in some types of lung cancer, the PI(3)K pathway activation through loss of inhibition due to *PTEN* mutations can cooperate with BRAF-dependent RTK signaling pathway activation to promote cancer development.

## Discussion

With deeper understanding of the underlying genetic mutations revolutionized by NGS technology, molecular testing has become an indispensable tool for lung cancer diagnosis^1,10^. In this study, we first performed an extensive performance evaluation of a NGS panel, OncoAim, on FFPE samples, then explored the genetic variants in Chinese NSCLC patients.

The turnaround time of the entire NGS process is about 2–3 days. We demonstrated that the targeted NGS had high sensitivity, specificity, accuracy and precision for both SNVs and INDELs. False-negative calls were predominantly low-frequency variants (MAF < 5%). Detection sensitivity rose up as sequencing coverage depth increased, up to 100% for MAF ≥ 5% variants at a certain coverage depth (500× for SNVs and 700× for INDELs) (Fig. 1A,B). High specificity was obtained across the whole sequencing coverage range (50-1000×). OncoAim test exhibited high concordance (94%) with ARMS-PCR approach for MAF ≥ 5% variants (Fig. 2), indicative of its high detection accuracy. Taken together, we concluded that the NGS-based OncoAim test had robust performance in FFPE samples. OncoAim panel covers common mutations (6000 hotspots in 59 cancer genes) in six prevalent Chinese cancer types (Oesophagus, stomach, liver, lung, breast and colon). Thus, in addition to lung cancer, it may be applied to other cancers. Further prospective studies of this panel on lung cancer and other cancer types should be performed to establish its application in clinical assay.

In five cases, *EGFR* exon 19 deletion was detected by ARMS-PCR, but not by NGS, which accounted for the major discordances observed between these two techniques. ARMS-PCR is super sensitive and can robustly identify alterations with 1% MAF^53^, whereas NGS can only reliably call variants with MAF ≥ 5%, particularly for INDELs^54^. Therefore, the difference in analytical sensitivity between these two methods may lead to inconsistencies in the test results. The low MAF could be caused by intratumoral heterogeneity, that is, the same tumor may possess cells that harbor different subclones with distinct mutations^55,56^.

We didn’t perform side-by-side comparison of OncoAim panel with previous genetic profiling tests. However, we noticed that most of our discoveries are consistent with previous reports^1,10^. For example, *EGFR*, *TP53*, *KRAS*, and *PIK3CA* were the top most frequently mutated genes in NSCLC. *EGFR* mutations were more common in female patients, and smokers tended to have higher mutation incidence of *KRAS*, and *EGFR* mutations were mutually exclusive with *KRAS* mutations. This validated the quality and effectiveness of our panel and bioinformatics pipeline, also reflected the robustness of these molecular signatures in NSCLC across populations with distinct demographic and racial background. In addition, our test sensitivity (>99% for variants with MAF ≥ 5% at >500X coverage depth) was comparable with that (95–99%) reported by Frampton, G.M.^54^.

We have also identified novel mutational patterns and novel correlations of genomic aberrations with patient characteristics in Chinese NSCLC. One intriguing finding is high mutation incidence of R213, G266 and R280 but low mutation incidence of G245 in *TP53* (Fig. 5B). These mutations together may aid in examining tumorigenesis, epidemiology, and therapeutic decisions of NSCLC in Chinese population^57^. The high frequency of *PTEN* A333*fs10 represents a previously undervalued genomic variant in Chinese NSCLC, and the future functional characterization of this variant for clinical diagnosis and drug development is well justified. For the first time, we revealed the significant correlation of mutations in 4 genes, including *EGFR*, *TP53*, *NFE2L2* and *PIK3CA*, with the specific histologic subtype of NSCLC in a single cohort study (Table 2), emphasizing the value of utilizing these molecular markers for subclassifying NSCLC patients and unearthing the distinct potential tumorigenesis mechanisms for NSCLC histologic subtypes. Moreover, OncoAim uncovered the previously unknown mutual exclusiveness of *NFE2L2* mutations with *EGFR* mutations, which highlighted the oncogenic nature of *NFE2L2*.

As a retrospective study using archived FFPE specimens, one caveat of the current study is that not all the patients’ clinicopathological information was available. The small number of patients in certain characteristic groups might limit the power of statistical analysis. For example, *BRAF* mutations are more prevalent in nonsmokers than in smokers (*P* = 0.019)^58^. Although we detected *BRAF* mutations only in nonsmokers, statistical analysis failed to show difference between nonsmokers and smokers (Table 2), likely because only 13 smokers existed in our cohort.

In summary, the present findings based on NGS test could aid in subdividing NSCLC patients according to specific molecular signatures, improving diagnosis and prognosis, and implementing precision and personalized treatment for Chinese NSCLC patients.

## Methods

### Ethics statement

This work has been approved by the West China Hospital Sichuan University Clinical Trials and Biomedical Ethics Committee (No. 2017-114). All methods were performed in accordance with the relevant guidelines and regulations and informed consent was obtained from all participants.

### Reference standards

We purchased 6 commercial reference standards (HD200, HD300, HD301, HD706, HD802, and HD260) from Horizon Diagnostics (Saint Louis, USA). These reference standards carry known mutation sites and mutation frequencies. Among them, HD200 is a Multiplex Reference Standard with known mutations in EGFR, KRAS, NRAS, PIK3CA and KIT (KIT Proto-Oncogene Receptor Tyrosine Kinase), and others are Gene-Specific Multiplex (HD260: EGFR V769_D770insASV Reference Standard; HD300: EGFR Gene-Specific Multiplex Reference Standard; HD706, EGFR V769_D770insASV Reference Standard; HD802, EGFR Gene-Specific Multiplex Reference Standard; HD301: KRAS Gene-Specific Multiplex Reference Standard).

### FFPE samples

FFPE samples of 452 NSCLC patients were collected from the archives of the following hospitals from 2013 to 2016 (Table 1): Peking University Third Hospital; Tongji Hospital, Tongji Medical College of Huazhong University of Science & Technology; Xijing Hospital, Fourth Military Medical University; and The First Affiliated Hospital, Zhejiang University. The inclusion criteria for this study are: (1) clear diagnosis of non-small cell lung cancer; (2) samples within 5 years; (3) tumor cell content ≥20%. A 4-μm section of a hematoxylin and eosin-stained slide was reviewed by a pathologist to ensure a sample volume ≥1 mm^3^, nucleated cellularity ≥80% or ≥30000 cells and tumor cell content ≥20%. Clinicopathological information was gathered for association analysis.

### DNA extraction, library preparation and sequencing

DNA of FFPE samples was extracted using the QIAamp DNA FFPE Tissue Kit (Qiagen, Hilden, Germany) strictly according to the manufacturer’s protocol. The extracted DNA was quantified using the Qubit dsDNA HS Assay kit and a Qubit 3.0 fluorimeter (Life Technologies). Libraries were constructed from 20 ng DNA and the OncoAim DNA Panel using the Ion AmpliSeq Library kit v2.0-96LV (Life technologies). The panel covers more than 6000 highly frequent mutation hotspots in 59 cancer genes (Table S1). Libraries were quantified with Qubit 3.0 fluorometer (Life technologies). The individual libraries were diluted and then pooled for generating an 8 pM library to amplify on Ion sphere particles (ISP) on the Ion One Touch 2 instrument (Life technologies). ISP templates were enriched, loaded on an Ion 318 chip and sequenced on the PGM sequencer (Life Technologies).

### Sequencing data analysis

OncoAim panel pipeline (OncoAim version 7.2) was used for sequencing data analysis. Briefly, the quality of raw reads (fastq files) was evaluated with FastQC (version 0.9.5, Babraham Bioinformatics, Cambridge, UK). High-quality reads were aligned against the human reference genome (hg19). The Burrows-Wheeler Aligner algorithm (https://github.com/lh3/bwa) was utilized for alignment, using default parameters. Insertions and deletions in sequence alignment files were left-aligned using a custom software tool, and left-aligned reads were processed using Freebayes (https://github.com/ekg/freebayes) for variant calling. The median coverage per locus was 500–1000x to ensure confident variant calling. The minimum mutation allele frequency (MAF) for SNVs and INDELs was set to 5%. Variants were annotated for effect prediction and clinical practice guidance. All the variants were manually checked on the Integrative Genomics Viewer (https://www.broadinstitute.org/software/igv/home).

### Performance validation

For SNV and indel validation, the six reference standards mentioned above were used. We mixed some of the reference standards by a 1:1 ratio to obtain more mutation sites and a wide MAF range. Then, individual standard and mixed standards were sequenced, targeting to >1000× coverage depth. Each bam file of sequencing data exported from the sequencer was randomly sampled (random selection of subsets of reads) to examine performance over a wide coverage depth range (50–1000×). We sampled 10 times for 50–500× and 3 times for 550–1000×. These sampled data were analyzed to identify variants.

For sensitivity analysis, all tested variants were assigned either a true positive (TP) if detected in the reference standards or false negative (FN) if not detected. Sensitivity at each test site was calculated as detected times/sampling times. For specificity analysis, each called variant was classified as a TP if the variant was a known mutation in the reference standards or a parental cell line, or a false positive (FP) if the variant was not a known mutation. Positive predictive value (PPV) was calculated as TP/ (TP + FP).

For accuracy analyses, we compared OncoAim panel with AmoyDx EGFR Mutations Detection Kit that uses the principle of Amplified Refractory Mutation System (ARMS) (Amoy Diagnostics, Xiamen, China). For inconsistent results, Sanger sequencing was performed using the specified primers (Table S2).

We validated test reproducibility by examining mutation calls in replicates of clinical FFPE specimens. The samples were analyzed 5 times (5 independent library preparations starting from the same extracted DNA) in three different experiments (including three replicates in a single run).

### Statistical analysis

The statistical analysis was done using R (R version 3.4.1). For two nominal variables, two-sided Fisher’s exact test was performed. For analysis involving more than 2 groups, following two-sided Fisher’s exact test, pairwise comparison with Bonferroni’s correction was performed. All tests were two-sided, and *P* < 0.05 was considered significant. Mutual exclusivity of variants was analyzed with R package ‘maftools’ (https://github.com/PoisonAlien/maftools), which performed Fisher’s exact test for mutual exclusive events.

## Supplementary information

Supplementary information

Dataset 1.

## Data Availability

All data generated or analyzed during this study are included in this article (and its Supplementary Information Files).
