# Supplementary material for: Validating a targeted next-generation sequencing assay and profiling somatic variants in Chinese non-small cell lung cancer patients
Source: Sci Rep. 2020 Feb 7;10:2070. doi: 10.1038/s41598-020-58819-5 (PMC7005734; doi:10.1038/s41598-020-58819-5)
Supplement: Supplementary file 1 — Supplementary information [file 41598_2020_58819_MOESM1_ESM.doc]

**SUPPLEMENTARY INFORMATION**

# Scientific Reports

Validating a targeted next-generation sequencing assay and profiling somatic variants in Chinese non-small cell lung cancer patients


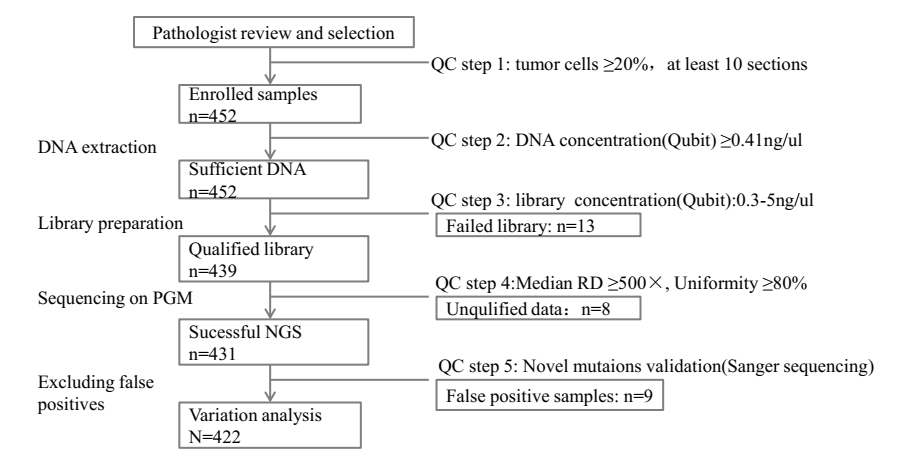
Ruirui Jiang^1, 2, 3^, Bo Zhang^4^, Xiaodong Teng^5^, Peizhen Hu^6^, Sanpeng Xu^7^, Zuyu Zheng^8^, Rui Liu^8^, Tingdong Tang^8^ and Feng Ye^2 *^

**Supplementary Fig. 1** Workflow for NGS profiling of the clininical NSCLC FFPE samples

**Supplementary Fig. 2** Gene mutation frequency in distinct gender group of patients. In each gender group, the mutation frequency was calculated by dividing the number of mutations in specific gene by the number of patients in that group.

**Supplementary Fig. 3** Gene mutation frequency in distinct age group of patients. The patient age was divided into 3 groups. In each age group, the mutation frequency was calculated by dividing the number of mutations in specific gene by the number of patients in that group.

**Supplementary Fig. 4** Gene mutation frequency in smoker or nonsmoker patient. In patient group with or without smoking history, the mutation frequency was calculated by dividing the number of mutations in specific gene by the number of patients in that group.

**Supplementary Fig. 5** Gene mutation frequency in patient with or without lymphatic spread. In each tumor group with or without lymphatic spread, the mutation frequency was calculated by dividing the number of mutations in specific gene by the number of patients in that group.

**Supplementary Fig. 6** Gene mutation frequency in patient with primary tumor or metastatic tumor. In the group of primary tumor or metastatic tumor, the mutation frequency was calculated by dividing the number of mutations in specific gene by the number of patients in that group.

**Supplementary Fig. 7** Gene mutation frequency in distinct histologic grades of tumor. In each histologic grade group, the mutation frequency was calculated by dividing the number of mutations in specific gene by the number of patients in that group.

**Supplementary Fig. 8** Gene mutation frequency in distinct histologic variants of tumor. In each histologic variant group, the mutation frequency was calculated by dividing the number of mutations in specific gene by the number of patients in that group.

**Supplementary Fig. 9** Gene mutation frequency in distinct tumor location. Tumor was divided into two locations: left lung and right lung. In each location group, the mutation frequency was calculated by dividing the number of mutations in specific gene by the number of patients in that group.

**Table S1** The list of 59 genes covered by OncoAim panel

**Table S2** Primers used for Sanger sequencing in the present study

| Gene | Exon | Primer |
| --- | --- | --- |
| EGFR | EGFR18F | GCTTTCCAGCATGGTGAGGGCT |
| EGFR | EGFR18R | GGGCTCCCCACCAGACCATG |

**Table S3a** Summary of known SNVs and original sampling data

Available in Supplementary_Table.xlsx

**Table S3b** Summary of sequencing metrics for reference standards used for assessment of SNV detection

| Sample ID | Total Reads | Mapped Reads | Mapping Rates | OnTarget Reads | OnTarget  Rate | Median RD | Uniformity |
| --- | --- | --- | --- | --- | --- | --- | --- |
| HD200 | 538767 | 537620 | 99.80% | 516378 | 96.05% | 1257 | 98.25% |
| HD200-HD706 | 498354 | 497085 | 99.75% | 476867 | 95.95% | 1160 | 98.10% |
| HD300 | 626923 | 625426 | 99.75% | 605392 | 96.80% | 1469 | 98.50% |
| HD300-HD706 | 536700 | 535502 | 99.80% | 515770 | 96.35% | 1246 | 97.85% |
| HD301 | 584555 | 583404 | 99.80% | 561501 | 96.25% | 1371 | 98.00% |
| HD301-HD706 | 435453 | 434617 | 99.80% | 418161 | 96.20% | 1025 | 97.70% |
| HD802 | 522058 | 520697 | 99.75% | 497471 | 95.55% | 1181 | 98.10% |
| HD802-HD260 | 495027 | 494103 | 99.80% | 472779 | 95.65% | 1083 | 98.70% |

**Table S3c** Summary of SNV detection performance (full sensitivity results)

Available in Supplementary_Table.xlsx

**Table S4** Summary of SNV detection performance (specificity)

| Sampling coverage | True positives | False positives | | Positive predictive value | |
| --- | --- | --- | --- | --- | --- |
|  |  | MAF≥ 5% | MAF< 5% | Mean | Confidence  interval |
| 1000× | 135 | 0 | 0 | 100.0% | 100.0% |
| 950× | 157 | 0 | 0 | 100.0% | 100.0% |
| 900× | 156 | 0 | 0 | 100.0% | 100.0% |
| 850× | 156 | 0 | 0 | 100.0% | 100.0% |
| 800× | 156 | 0 | 0 | 100.0% | 100.0% |
| 750× | 157 | 0 | 0 | 100.0% | 100.0% |
| 700× | 155 | 0 | 0 | 100.0% | 100.0% |
| 650× | 152 | 0 | 0 | 100.0% | 100.0% |
| 600× | 152 | 0 | 0 | 100.0% | 100.0% |
| 550× | 148 | 0 | 0 | 100.0% | 100.0% |
| 500× | 486 | 0 | 0 | 100.0% | 100.0% |
| 450× | 471 | 0 | 0 | 100.0% | 100.0% |
| 400× | 463 | 0 | 0 | 100.0% | 100.0% |
| 350× | 437 | 0 | 0 | 100.0% | 100.0% |
| 300× | 418 | 0 | 0 | 100.0% | 100.0% |
| 250× | 383 | 0 | 0 | 100.0% | 100.0% |
| 200× | 334 | 0 | 0 | 100.0% | 100.0% |
| 150× | 285 | 0 | 0 | 100.0% | 100.0% |
| 100× | 221 | 0 | 0 | 100.0% | 100.0% |
| 50× | 160 | 0 | 0 | 100.0% | 100.0% |

.

**Table S5a** Summary of known INDELs and original sampling data

Available in Supplementary_Table.xlsx

**Table S5b** Summary of sequencing metrics for reference standards used for assessment of INDEL detection

| Sample ID | Total Reads | Mapped Reads | Mapping Rates | OnTarget Reads | OnTarget  Rate | Median RD | Uniformity |
| --- | --- | --- | --- | --- | --- | --- | --- |
| HD200 | 538767 | 537620 | 99.80% | 516378 | 96.05% | 1257 | 98.25% |
| HD200-HD706 | 498354 | 497085 | 99.75% | 476867 | 95.95% | 1160 | 98.10% |
| HD300 | 626923 | 625426 | 99.75% | 605392 | 96.80% | 1469 | 98.50% |
| HD300-HD706 | 536700 | 535502 | 99.80% | 515770 | 96.35% | 1246 | 97.85% |
| HD802 | 522058 | 520697 | 99.75% | 497471 | 95.55% | 1181 | 98.10% |
| HD802-HD260 | 495027 | 494103 | 99.80% | 472779 | 95.65% | 1083 | 98.70% |

**Table S5c** Summary of INDEL detection performance (full sensitivity results)

Available in Supplementary_Table.xlsx

**Table S6** Summary of INDEL detection performance (specificity)

| Sampling coverage | True positives | False positives | | Positive predictive value | |
| --- | --- | --- | --- | --- | --- |
|  |  | MAF≥5% | MAF< 5% | Mean | Confidence interval |
| 1000× | 19 | 0 | 3 | 86.4% | 72.0-100% |
| 950× | 18 | 0 | 3 | 85.7% | 70.7-100% |
| 900× | 19 | 0 | 2 | 90.5% | 77.9-100% |
| 850× | 18 | 0 | 2 | 90.0% | 76.9-100% |
| 800× | 17 | 0 | 1 | 94.4% | 83.9-100% |
| 750× | 16 | 0 | 0 | 100% | 100% |
| 700× | 17 | 0 | 0 | 100% | 100% |
| 650× | 16 | 0 | 0 | 100% | 100% |
| 600× | 15 | 0 | 0 | 100% | 100% |
| 550× | 14 | 0 | 0 | 100% | 100% |
| 500× | 47 | 0 | 0 | 100% | 100% |
| 450× | 42 | 0 | 0 | 100% | 100% |
| 400× | 42 | 0 | 0 | 100% | 100% |
| 350× | 40 | 1 | 0 | 97.6% | 92.8-100% |
| 300× | 40 | 0 | 0 | 100% | 100% |
| 250× | 38 | 0 | 0 | 100% | 100% |
| 200× | 36 | 0 | 0 | 100% | 100% |
| 150× | 30 | 0 | 0 | 100% | 100% |
| 100× | 16 | 0 | 0 | 100% | 100% |
| 50× | 1 | 0 | 0 | 100% | 100% |

**Table S7** Summary of NGS/ARMS-PCR discordances

| **NGS+/ARMS- discordances** | | | |
| --- | --- | --- | --- |
| Sample | NGS EGFR | ARMS-PCR | Probable discordance reason |
| H50 | p.E709A、 p.G719S | Not detected | ARMS miscall |

| **NGS-/ARMS+ discordances** | | | |
| --- | --- | --- | --- |
| Sample | NGS EGFR | ARMS-PCR | Probable discordance reason |
| H16 | Not detected | 19-Del | Sensitivity/Tissue heterogeneity |
| H26 | Not detected | 19-Del | Sensitivity/Tissue heterogeneity |
| H81 | Not detected | 19-Del | Sensitivity/Tissue heterogeneity |
| H34 | Not detected | 19-Del | Sensitivity/Tissue heterogeneity |
| H47 | Not detected | 19-Del | Sensitivity/Tissue heterogeneity |
| H48 | Not detected | G719X | Sensitivity/Tissue heterogeneity |

**Table S8** Precision (reproducibility and repeatability) evaluated for 3 FFPE tumor samples

| Sample | Mutations | NGS results | | | | |
| --- | --- | --- | --- | --- | --- | --- |
|  |  | Rep 1 | Rep 2 | Rep 3 | Rep 4 | Rep 5 |
| 1 | KRAS p.G12V | 0.131 | 0.127 | 0.132 | 0.109 | 0.132 |
| 2 | KRAS p.G12D | 0.26 | 0.176 | 0.307 | 0.257 | 0.186 |
|  | TP53 p.R248W | 0.078 | 0.118 | 0.152 | 0.109 | 0.126 |
| 3 | TP53 p.R333fs*12 | 0.297 | 0.304 | 0.312 | ND | 0.257 |
|  | EGFR p.L858R | 0.202 | 0.233 | 0.217 | 0.227 | 0.232 |

ND, not detected; Rep: replicate. Overall concordance was calculated as the average of pair-wise replicate concordances. Pair-wise replicate concordance was calculated as (2 x # of concordant alterations detected)/(# of alterations detected in replicate 1 + # of alterations detected in replicate 2).
